# P03-008 - Gastrointestinal involvement in Behçet’s syndrome

**DOI:** 10.1186/1546-0096-11-S1-A203

**Published:** 2013-11-08

**Authors:** I Hatemi, G Hatemi, Y Erzin, AF Celik, H Yazici

**Affiliations:** 1 Gastroenterology, Istanbul University, Cerrahpasa Medical School, Istanbul, Turkey; 2 Rheumatology, Istanbul University, Cerrahpasa Medical School, Istanbul, Turkey

## Introduction

Gastrointestinal involvement in Behçet's syndrome (BS) can be a severe complication resulting in perforation and massive bleeding. Controlled data regarding treatment is lacking and long term prognosis is not well known.

## Objectives

To report the demographic and disease characteristics, type of involvement, treatment modalities and outcome of BS patients with gastrointestinal involvement (GIBS).

## Methods

We retrospectively reviewed the charts of all BS patients evaluated with a suspicion of gastrointestinal involvement. We identified those with GIBS and surveyed their demographic features, other BS manifestations, clinical, endoscopic and histologic gastrointestinal findings, and treatment modalities. Patients were evaluated either in the outpatient clinic or if not possible by phone calls to assess their outcome.

## Results

Among the 8058 recorded BS patients in our multidisciplinary outpatient clinic, 69 had symptoms suggesting gastrointestinal involvement and lesions on endoscopy. Among these, 18 patients had other reasons for their gastrointestinal symptoms and endoscopic lesions. The remaining 51 patients had GIBS. The presenting symptoms were acute abdomen caused by perforations in 4/51 patients, massive bleeding in 8/51 patients and abdominal pain and/or diarrhea in 39/51 patients. Surgery had to be performed in 20/51 patients, and 4 of them had to be re-operated for development of stricture, progressive disease, relapse, and corrective surgery, 1 patient each. The most commonly used drugs for initial management were azathioprine 2-2.5 mg/kg/day (n=33) and 5 ASA compounds 3-4 g/day (n=13). Remission was observed and there were no relapses during a mean follow-up of 44.3±46.9 months in 22/33 (67%) patients who had initially been prescribed azathioprine (2.5 mg/kg) and during 45.0±50.1 months in 9/13 (68%) patients who had been prescribed 5 ASA compounds. Other than the 33 patients who used azathioprine as their initial treatment, remission was also obtained with azathioprine in 3/4 patients who were resistant to 5 ASA compounds. Among the 10 patients who had relatively severe symptoms and persistent large ulcers despite at least 6 months of azathioprine treatment, endoscopic and symptomatic remission could be obtained with thalidomide in 4 patients, infliximab in 4 patients and adalimumab in 2 patients. After a mean follow-up of 7.1± 4.8 years (range 0.25 – 17 years), 42 (84%) patients were in remission and 14 (28%) of these were off treatment. Four (8%) patients were still active, 3 (6%) patients had died due to non-GI releated reasons and 2 (4%) were lost to follow-up. The reasons for death were pulmonary artery thrombosis, infection and acute renal failure due to amyloidosis in 1 patient each.

## Conclusion

84% of patients with GIBS were in remission after a mean of 7 years of follow-up. Surgery was required in 40% of patients with GIBS. 5 ASA compounds or azathioprine provided remission and prevented relapses in two thirds of the patients .The latter was also beneficial in some patients resistant to 5 ASA compounds. Resistant and relapsing cases could be managed with thalidomide or TNF-alpha antagonists.

## Competing interests

None Declared.

